# Safety and efficacy of acupuncture for the treatment of chronic obstructive pulmonary disease: A systematic review protocol

**DOI:** 10.1097/MD.0000000000017112

**Published:** 2019-09-13

**Authors:** Mingxia Yu, Longxia Gao, Yanhua Kong, Yue Yan, Qi Shi, Dongxu Si, Haipeng Bao, Huizhuo Sun, Lei Li, Youlin Li

**Affiliations:** a Beijing University of Chinese Medicine, Beijing,; b Inner Mongolia Autonomous Region Hospital of Traditional Chinese Medicine, Hohhot,; c The 2nd Department of Pulmonary Disease in TCM, The Key Unit of SATCM Pneumonopathy Chronic Cough and Dyspnea, Beijing Key Laboratory of Prevention and Treatment of Allergic Diseases with TCM (No. BZ0321), Center of Respiratory Medicine, China-Japan Friendship Hospital; National Clinical Research Center for Respiratory Diseases, Beijing, China.

**Keywords:** acupuncture, chronic obstructive pulmonary disease, protocol, systematic review

## Abstract

**Background:**

Chronic obstructive pulmonary disease (COPD) is a common chronic respiratory disease with increasing morbidity and mortality that cause huge social and economic loss. Although recommended by guidelines, pulmonary rehabilitation has not been widely applied in clinics because of its inherent limitations. Acupuncture therapy (AT) as one of the most popular treatments in traditional Chinese medicine has been used to treat COPD. We aim to evaluate the safety and efficacy of acupuncture in the treatment of COPD.

**Methods:**

Web of science, PubMed, Springer, Medline, Cochrane Library, EBASE, WHO International Clinical Trials Registry Platform (ICTRP), China National Knowledge Infrastructure Database (CNKI), Wan Fang Database, Chinese Scientific Journal Database (VIP), and Chinese Biomedical Literature Database will be searched from their inception to May 10, 2019. Randomized controlled trials that evaluated the safety and efficacy of acupuncture for the treatment on patients with COPD will be included. The primary outcome measures will include Dyspnea scores, lung function and blood eosinophils. The secondary outcome measures will include St George's Respiratory Questionnaire and 6-minute walk distance. Study selection, data extraction, and risk of bias assessment will be independently undertaken, respectively. Statistical analysis will be conducted by RevMan software (version 5.3).

**Results:**

This study will provide high-quality synthesis based on current evidence of acupuncture treatment for COPD in several aspects, including symptom score, quality of life score, side effects and laboratory examination, such as lung function text, blood eosinophils (EOS) etc.

**Conclusion:**

The results of this study will provide updated evidence for weather acupuncture is an effective and safe intervention for COPD.

**Ethics and dissemination:**

It is not necessary for this systematic review to acquire an ethical approval. This review will be disseminated in a peer-reviewed journal or conference presentation.

**PROSPERO registration number:**

PROSPERO CRD42019136087.

## 1 Introduction

Chronic obstructive pulmonary disease (COPD) is characterized by persistent respiratory symptoms and airflow limitation that is due to airway and/or alveolar abnormalities usually caused by significant exposure to cigarette smoking or other noxious particles, gases.^[1]^ Dyspnea, Cough, Sputum production, Wheezing, and chest tightness are the most frequent symptoms, in addition, Fatigue, weight loss, and anorexia are common in patients with more severe forms of COPD.^[1–3]^ It was reported that COPD is the fourth leading cause of death worldwide and is predicted to be the third by 2030.^[2,3]^ With the increasing prevalence of smoking in developing countries and aging populations in high-income countries, COPD is expected to worsen over the next 30 years, and over 4.5 million annual deaths caused by COPD are predicted to occur in 2030.^[4,5]^ One study found that the overall prevalence of spirometry-defined COPD was 8.6%, accounting for 99.9 million people with COPD in China.^[6]^ COPD brings a significant burden not only for patients and their families but also for social healthcare systems in general.^[4,7]^

COPD cannot be completely cured. At present, the treatment of the COPD is mainly included Inhaled combination therapy oral prophylactic antibiotic therapy, long-term oxygen therapy, ambulatory and short-burst oxygen therapy, and managing pulmonary hypertension and Cor Pulmonale. Pharmacological intervention, such as beta2-agonists and Inhaled corticosteroids (ICS), could relieve symptoms, reduce acute exacerbations. However, both systemic and local side effects of them have been reported, such as oropharyngeal candidiasis and hoarseness, and risk of pneumonia.^[8,9]^

Acupuncture therapy (AT) as one of the most popular treatments in traditional Chinese medicine (TCM) has been widely used to treat a variety of diseases such as allergic rhinitis,^[10]^ asthma,^[11]^ breast cancer patients.^[12]^ A certain operation method can be used to achieve the treatment through the conduction of meridians and acupoints. With few side effects, wide indication, convenient operation, economical, and safety, acupuncture has been used for thousands of years in China. It may be effective in improving functional effects and quality of life in COPD patients. Besides, ATs may also improve pulmonary function and nutritional state of patients with COPD.^[13,14]^ It is reported that ATs may contributed to the reduction of COPD-related dyspnea,^[15]^ and recent systematic review of randomized controlled trials of ATs for treating COPD found that ATs may result in clinically important.^[16]^ In this review, we aim to systematically assess the efficacy and safety of acupuncture for COPD.

## 2 Methods

### 2.1 Study registration

This systematic review protocol has been registered on PROSPERO as CRD42019136087. Available from: http://www.crd.york.ac.uk/PROSPERO/display_record.php?ID=CRD42019136087. The protocol follows the Cochrane Handbook for Systematic Reviews and Meta-Analysis Protocol (PRISMA-P) statement guidelines.^[17]^ We will describe the changes in our full review if needed.

### 2.2 Inclusion criteria for study selection

#### 2.2.1 Type of studies

In order to evaluate the efficacy and safety of acupuncture in the treatment of COPD, all relevant randomized controlled trials (RCTs) and cohort studies (CS) published in English and Chinese on acupuncture for COPD can be included. Non-RCTs, reviews, case report, experimental studies, expert experience, and duplicated publications will be excluded.

#### 2.2.2 Type of participants

Study participants in different age ranges with a confirmed diagnosis of COPD can be included in the study without restricting nationality, sex, race, occupation, or education. The diagnostic criteria of COPD are in accordance with the Global Initiative for Chronic Obstructive Lung Disease (GOLD) guidelines,^[18]^ and those who had an acute exacerbation within 4 weeks before the study were excluded. Patients with a diagnosis of asthma, cystic fibrosis, bronchiectasis, or other lung diseases, cardiovascular disease, renal failure, thyroid dysfunction, hepatic function disorder, cancer, and severe mental disorder were excluded.

#### 2.2.3 Types of interventions

##### 2.2.3.1 Experimental interventions

The experimental group treated with only acupuncture including manual acupuncture, electroacupuncture, ear acupuncture, scalp acupuncture, plum blossom needle, fire needling, or dermal needle, regardless of the number of acupoints, the method of needle insertion, duration and frequency. The studies involved moxibustion, laser acupuncture, transcutaneous electrical nerve stimulation, pharmaco-acupuncture, or acupressure would be excluded. De qi (tingling, numbness, and heaviness) was achieved during the manipulation of needles at every point in the real acupuncture group.

##### 2.2.3.2 Control interventions

The control group treated with no treatment or placebo acupuncture (a special needle with blunt tip does not penetrate the skin, but a small pricking sensation is felt by the patient), or sham acupuncture (the tips of the sham needles were blunt and appeared to be penetrating the skin but actually telescoped back into place in the sham acupuncture group and the needles were not inserted into the skin),^[19]^ and other interventions (e.g., medicine, moxibustion, point application, oxygen therapy, and other physical interventions).

#### 2.2.4 Types of outcome measures

Primary outcomes

1. Symptom score will be assessed as the primary outcome. Dyspnea scores, according to the COPD assessment test (CAT) criteria, modified Medical Research Council (m MRC) criteria,^[20]^ and or the Borg category.
2. The pulmonary function change in forced expiratory volume in 1 second and change in forced ventilatory capacity25 (trough, peak and, average) and other measures of pulmonary function.
3. Biomarkers in COPD: blood eosinophils^[18]^
4. Side effects and adverse events

Secondary outcomes

The secondary outcomes of this review mainly include the following aspects:

1. The quality of life of the patients was measured with St. George's Respiratory Questionnaire (SGRQ)^[21]^ and the Chronic Respiratory Disease Questionnaire (CRDQ or CRQ).^[22]^
2. Exercise tolerance: for example, 6 minute walk test, 30 shuttle walk test.
3. Supplementary examination (e.g., sputum smear)
4. Exacerbations

### 2.3 Search methods for identification of studies

#### 2.3.1 Electronic searches

The databases we will search, consists of the English databases and the Chinese databases. The English databases are Web of science, PubMed, Springer, Medline, Cochrane Library, EBase, WHO International Clinical Trials Registry Platform (ICTRP), and the Chinese databases are China National Knowledge Infrastructure Database (CNKI), Wan Fang Database, Chinese Scientific Journal Database (VIP), and Chinese Biomedical Literature Database. These databases were searched from their inception to 30 April 2019.

#### 2.3.2 Searching other resources

The relevant systematic reviews, reference list of studies and conference abstracts will be searched. In addition, we will search potential gray literature in Open Grey.eu. Recently completed studies and ongoing studies will be searched.

#### 2.3.3 Searching strategy

The search strategy for Web of science is listed in Table 1, which includes all search terms, this search strategy will be modified as required for other electronic databases.

**Table 1:**
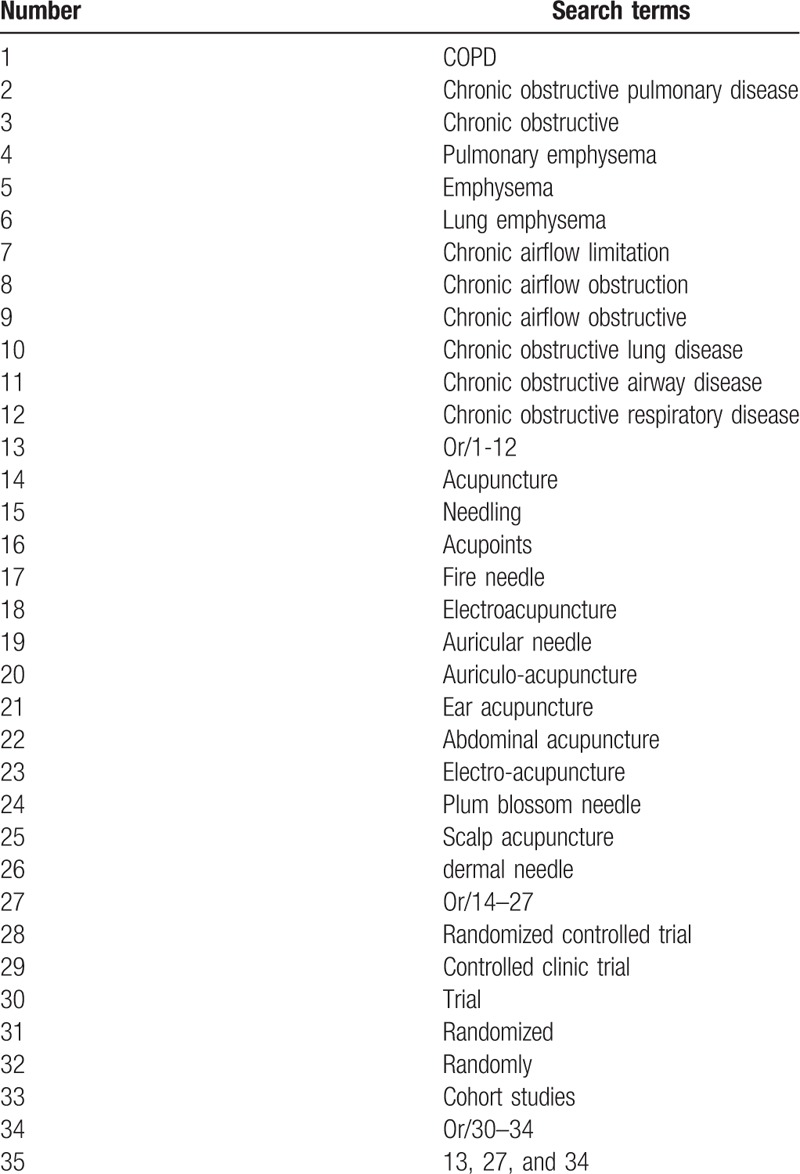
Web of science search strategy.

### 2.4 Data collection and analysis

#### 2.4.1 Date collection

**Figure 1:**
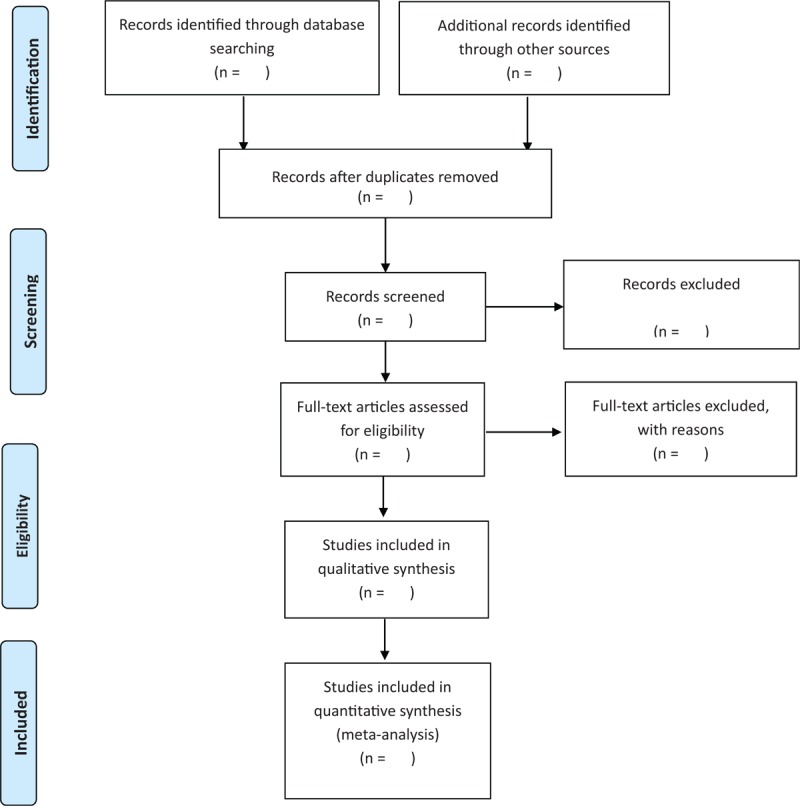
The Systematic Reviews and Meta-Analysis Protocol flow chart of the selection process.

All review authors have received Search items training to ensure a good understanding of the purpose and process of the review. Two review authors (DS and LG) independently screened the literature, extracted the data and cross-checked them. In case of any disagreement, a third party was consulted for assistance. The missing dates should be supplemented by contacting the author. In literature selection, the thesis title and abstract were read first, and then the full text was further read after excluding obviously irrelevant literatures to determine whether the final inclusion was made. The study of screening flow diagram is summarized in Figure 1.

Data extraction mainly includes:

(1) The basic characteristics of the study (1st author, time of publication, source/journal, and country);
(2) participant characteristics (sample size, average age, gender, inclusion, and exclusion criteria, baseline situation);
(3) Interventions (type of acupuncture, randomization, allocation concealment, and blinding methods);
(4) Key elements of risk assessment of bias
(5) Outcomes (measures, main outcomes, adverse effects, and follow up).

#### 2.4.2 Assessment of risk of bias

The risk of bias in each study will be assessed by 2 independent authors (SX and CL) using the criteria outlined in the Cochrane Handbook for Systematic Reviews of Interventions. If the number of included studies is more than 10, we will generate funnel plots to detect reporting biases and small-study effects.

#### 2.4.3 data analysis

Data analysis was performed using RevMan5.3.5 software provided by the Cochrane Collaboration. Subgroup analysis will be carried out according to the different types of ATs, characteristics of participants, and outcome measures. A risk ratio (RR) with 95% confidence intervals (CIs) will be used to estimate the dichotomous outcomes, and the continuous data will be analyzed by mean difference (MD) or standard MD (SMD)with 95%CIs. We will assess heterogeneity by visually inspecting the forest plots to detect nonoverlapping CIs and by investigatingX^2^ (with *P* value >.10 indicating no heterogeneity) and *I*^2^ statistic. *I*^2^ ≥ 50% will be considered as representing substantial heterogeneity and the random effect model is adopt to analyze, while *I*^2^ < 50% will be taken as evidence of no heterogeneity and the fixed effect model will be used for statistics.

#### 2.4.4 Subgroup analysis

Subgroup analysis will be carried out based on different types of ATs, characteristics of participants, and outcome measures.

#### 2.4.5 Sensitivity analysis

We will take sensitivity analyses to test the robustness and reliability of the results, if the dates are sufficient. Sensitivity analysis mainly focuses on research characteristics or types, such as methodological quality, and the effects on total effect are examined by excluding certain low-quality studies or non-blinded studies.

## 3 Discussion

At present, It is reported that inhaled beta-agonists were associated with an increased risk of heart failure hospitalization, and all-cause mortality.^[23]^ It is also reported that the use of β2 agonists, may lead to excessive tachycardia, peripheral vasodilation, hypokalemia especially in conjunction with diuretics, QTC prolongation,^[24,25]^ and myocardial ischemia.^[26]^ Some previous reports suggested that beta-blockers are properties only in mild to moderate COPD, with this positive effect being eliminated in more severe airway disease. Glucocorticoids can used to solve the more severe airway disease. However, long-term use of glucocorticoids may have some side-effects, such as toxicity, dependence, and may lead to financial strain due to the cost of long-term treatment.^[27,28]^ Although pharmacological therapy has a good effect in improving symptoms, the course of disease is longer, the condition is easy to repeat and the side effects are relatively large. Therefore, no-drug interventions are urgently needed to be promoted to alleviate clinical symptoms and reduce the risk of side effects.

As a complementary and alternative medical method based on the theory of TCM, acupuncture has been used in China for thousands of years. It is reported that acupuncture can improves pulmonary function of cats with COPD by down-regulating inflammatory reaction and expression of macrophage migration inhibitory factor/CD 74-CD 44/p 38 MAPK signaling in lung tissues.^[29]^ One study reported that acupuncture intervention improved pulmonary function via promoting immunoregulation in COPD rats.^[30]^ It also suggested that acupuncture regulates inflammatory cytokines and contributes to lung protection in a rat model of smoke-induced COPD by modulating HDAC2.^[31]^ Actually, AT has been used to treat patients with COPD in China.^[32]^ Although the mechanism in still unclear, results of clinical studies indicates that AT has a comparable effect to the medication treatment on patients with moderate to severe COPD and it is safe with few side effects.^[33]^ We hope this systematic review will provide more reliable evidence to help patients and clinicians in the management of COPD.

In this systematic review, we set experimental group treated with acupuncture including manual acupuncture, electroacupuncture, ear acupuncture, scalp acupuncture, plum blossom needle, fire needling, or dermal needle, regardless of the number of acupoints, the method of needle insertion, duration and frequency. We also select the all relevant RCTs and CS published in English and Chinese on acupuncture for COPD. In addition, we regard the blood eosinophils as one of the primary outcomes.

However, there are limitation, such as the language only include Chinese and English. Moreover, different types of acupuncture, acupoints, duration, frequency, the age of patients, and degree of COPD may cause high heterogeneity.

## Author contributions

**Conceptualization:** Mingxia Yu, Youlin Li.

**Data curation:** Dongxu Si, Haipeng Bao, Huizhuo Sun, Lei Li.

**Formal analysis:** Mingxia Yu, Longxia Gao.

**Project administration:** Mingxia Yu, Youlin Li.

**Supervision:** Yanhua Kong, Yue Yan, Qi Shi.

**Writing – original draft:** Mingxia Yu.

**Writing – review & editing:** Mingxia Yu, Youlin Li.
